# Photon-Trapping Microstructure for InGaAs/Si Avalanche Photodiodes Operating at 1.31 μm

**DOI:** 10.3390/s22207724

**Published:** 2022-10-12

**Authors:** Hewei Zhang, Yang Tian, Qian Li, Wenqiang Ding, Xuzhen Yu, Zebiao Lin, Xuyang Feng, Yanli Zhao

**Affiliations:** Wuhan National Laboratory for Optoelectronics, Huazhong University of Science and Technology, Wuhan 430074, China

**Keywords:** InGaAs/Si avalanche photodiodes, F-P resonant cavity, photon trapping

## Abstract

With the rapid development of photo-communication technologies, avalanche photodiode (APD) will play an increasingly important role in the future due to its high quantum efficiency, low power consumption, and small size. The monolithic integration of optical components and signal processing electronics on silicon substrate chips is crucial to driving cost reduction and performance improvement; thus, the technical research on InGaAs/Si APD is of great significance. This work is the first to demonstrate the use of a photon-trapping (PT) structure to improve the performance of the InGaAs/Si APD based on an SOI substrate, which exhibits very high absorption efficiency at 1310 nm wavelength while the thickness of the absorption layer is kept at 800 nm. Based on the optical and electrical simulations, an optimized InGaAs/Si PT-APD is proposed, which exhibits a better performance and a higher responsivity compared to the original InGaAs/Si APD.

## 1. Introduction

As high-speed communication technology continues to grow exponentially, optical communication links are used in data centers to handle higher capacity data traffic and bandwidth, and high sensitivity avalanche photodetectors (APDs) are playing an increasingly important role in optical communication links [1,2]. Existing mature commercial APD devices have some inherent disadvantages, group III-V material devices such as InGaAs/InP APD, due to their larger ratio of impact ionization coefficients than Si, limit their excess noise and bandwidth performance [3,4]. For Ge/Si APD, the absorption properties of Ge materials in the optical communication band are smaller than those of direct bandgap semiconductor materials such as InGaAs, and the electron mobility of Ge and Si materials are lower than that of III-V compounds, so their performance also has room for further improvement. InGaAs/Si APD combines the advantages of both types of materials; this kind of APD device fabricated by the heterojunction of Si and InGaAs can combine the advantages of the long response wavelength and high-absorption coefficient of InGaAs, with the low excess noise and good temperature characteristic of Si material. Therefore, there is an opportunity to obtain detectors with high quantum efficiency, a small dark current, and a high-gain bandwidth product [5]. Moreover, compared with APD made of III-V compounds, InGaAs/Si APD can be integrated on Silicon On Insulator (SOI) substrate, which is effectively compatible with today’s integrated circuit industry. Another advantage is the optical limiting characteristics of SOI substrate, which is equivalent to indirectly improving the quantum efficiency of the APD device.

Previously, InGaAs materials were grown on Si by epitaxy, but the dislocations and defects of this material are very large, which cannot meet requirements. Bonding the InGaAs material to Si material to form a new InGaAs/Si heterojunction material is the best way to develop an InGaAs/Si APD with excellent performance [6]. In contrast to the usual MOCVD and MBE growth methods, the bonding technique can be unaffected by the crystal orientation and lattice matching of the heterojunction material. In 2020, InGaAs/Si APD was prepared by Wanhua Zheng et al. through low-temperature bonding technology, which provided a technical reference for the preparation of a new generation of high-performance APD [7].

Enhancing light absorption of photodetectors by resonant cavity effect or light-trapping structure is a hot research direction recently. In 2012, Sheng-Di Lin et al. proposed and demonstrated a novel device structure of resonant cavity-enhanced photodetector (RCE-PD), they adjusted the effective cavity length of the RCE-PD by changing the fill factor of the two-dimensional grating [8]. In 2017, M. Saif Islam et al. improved the absorption efficiency of Si photodetectors with a thin intrinsic layer thickness of fewer than 2 μm by an order of magnitude through etching micro- and nano-holes, which is called a photon-trapping (PT) structure; further optimization can increase the absorption efficiency to more than 70% [9]. In 2020, Chuan Seng Tan et al. introduced photon-trapping micro-structures into a GeSn/Ge-based photodetector for the first time, achieving a four-fold photo-response enhancement at 2 µm, and the results also showed that the introduction of photon-trapping micro-structures can reduce the dark current density of the device [10]. However, to date, most researchers have applied PT structure to Ge/Si or Si detectors, and there are few studies on using PT structure to enhance the absorption properties of InGaAs/Si APD based on an SOI wafer [9,11,12]. The proposal of this study can reduce the thickness of the intrinsic layer to improve the response rate while achieving a high responsivity that Ge/Si APDs cannot achieve in the optical communication band.

In this work, an InGaAs/Si APD enhanced by the PT structure was proposed for the first time, the optical simulation model mainly refers to the stack structure developed by Wanhua Zheng et al. [7]. The main advantage of introducing the PT structure into InGaAs/Si APD is that the absorption efficiency near the target wavelength of 1310 nm can be increased to about 96%, while the thickness of the absorption layer is kept at a thin 800 nm thickness. This work explores a new possibility for high-sensitivity receivers that can be used in long-wavelength optical communication receiving systems [13,14]. 

## 2. The Problem Geometry and Simulation Method

The detector we designed is a novel InGaAs/Si photon-trapping APD (PT-APD) that enhances the responsivity through the photon-trapping nano-hole array structure. The PT structure consists of periodic nano-holes in a square lattice located at the center of the device. The PT structure can be fabricated on a vertical InGaAs-Si APD, which allows the light field distribution of the device to be modified by adjusting the PT-structure parameters, such as the period, depth, and diameter of the holes [9,15,16]. The substrate uses a SOI wafer to provide the necessary high-refractive-index difference to confine photons in the active layer of the detector.

**Figure 1:**
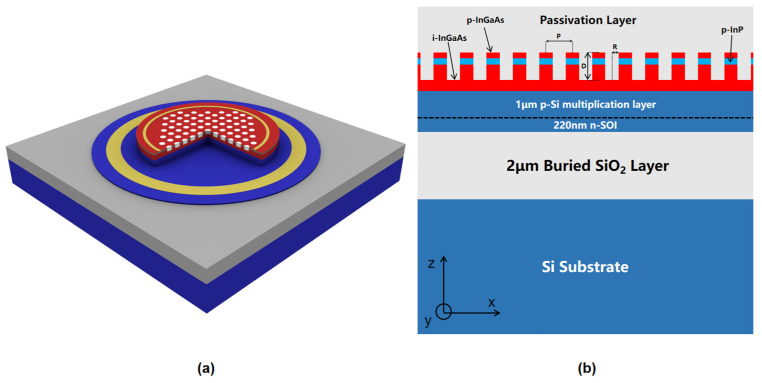
(**a**) 3D schematic of InGaAs/Si PT-APD; (**b**) cross-sectional diagram of the InGaAs/Si PT-APD.

The stack structure of the device from top to bottom is as follows: 0.15 μm SiO_2_ passivation layer, 0.1 μm p-InGaAs layer, 0.1 μm p-InP layer, 0.8 μm i-InGaAs absorber layer, 1 μm p-Si multiplication layer, 220 nm n-SOI layer, 2 μm buried oxide layer, and Si substrate. The doping levels are 5 × 10^19^ cm^−3^ (p-InGaAs), 1 × 10^18^ cm^−3^ (p-InP), 1 × 10^15^ cm^−3^ (i-InGaAs), 2 × 10^16^ cm^−3^ (p-Si), and 1 × 10^19^ cm^−3^ (n-SOI), respectively. The passivation layer should be kept thick enough to cope with the high dark current that may be caused by etching, and the i-InGaAs absorber layer should be set to 800 nm to maintain a balance between high absorption and high-speed response. The envisioned novel InGaAs/Si PT-APD design is shown in Figure 1a, the nano-holes are filled with SiO_2_ dielectric, and the n-SOI and p-InGaAs layers are coated with ring electrodes; the double mesa structure of the device is designed to reduce the parasitic capacitance. The period of the nano-hole array structure is set to *P*, the etching depth of the nano-hole is set to *D*, the etching radius of the nano-hole is set to *R*, and the absorption efficiency is defined as the ratio of the light power absorbed by the intrinsic absorption layer to the incident optical power.

We briefly describe one fabrication method of this novel InGaAs/Si APD: The SOI and InGaAs/InP wafers are cleaned by physical and chemical methods to remove the dirt on the surface [17,18]. The epitaxial structures of InGaAs/InP wafer is made in the following process: the p^+^-InGaAs (0.1 μm, 5 × 10^19^ cm^−3^) layer was epitaxial on the n-InP substrate, then a layer of p-InP (0.1 μm, 1 × 10^18^ cm^−3^) and a 0.8-μm layer of intrinsic InGaAs layer is followed. The two wafers are put into the vacuum bonding machine and are bonded at a certain temperature and pressure. By optimizing the cleaning methods and bonding conditions, Si/InGaAs can be directly bonded at a low temperature. Next, the InP substrate was thinned and etched to remove, and the mesa structure of APD was obtained by step-photo lithography [7]. Electron beam lithography (EBL) and inductively coupled plasma (ICP) techniques are mainly used to fabricate PT structures, E-beam evaporation is used to deposit metal electrodes to form metal-semiconductor contacts. On the top of the wafer, SiO_2_ was deposited using plasma-enhanced chemical vapor deposition to protect the metal electrodes and passivate the etched sidewall [11].

In terms of research methods, we simulated through ANSYS Lumerical FDTD software, which is based on the finite-difference time-domain algorithm. In this simulation model, the periodic boundary condition is applied in the X and Y directions, the Perfect Matching Layers (PML) boundary condition is used to terminate the propagation of the electromagnetic field in the Z direction. In addition, the simulation data uses the refractive index of Si, InP, and InGaAs [19,20], and the refractive index of SiO_2_ is set to 1.45. The i-InGaAs layer is the region where we record the absorbed light power, and the light source is set to TE polarization (the polarization direction is perpendicular to the Y-axis).

In addition to conducting optical studies, we performed electrical simulation on the designed devices through the CHARGE module based on the carrier drift-diffusion equation and Poisson equation. The simulation also considers the carrier recombination, including the Shockley–Read–Hall recombination, Auger recombination, radiation recombination, impact ionization, and band-to-band tunneling; the photocurrent of the APD is obtained by importing the photo-generated carrier velocity from the optical simulation into the electrical model. In this electrical model, the positive and negative electrodes are set as aluminum electrodes, the sampling interval of the reverse bias voltage is −1 V, and the simulation step size gradually decreases as the reverse bias voltage gets closer to the breakdown voltage.

## 3. Structure Design and Discussion

**Figure 2:**
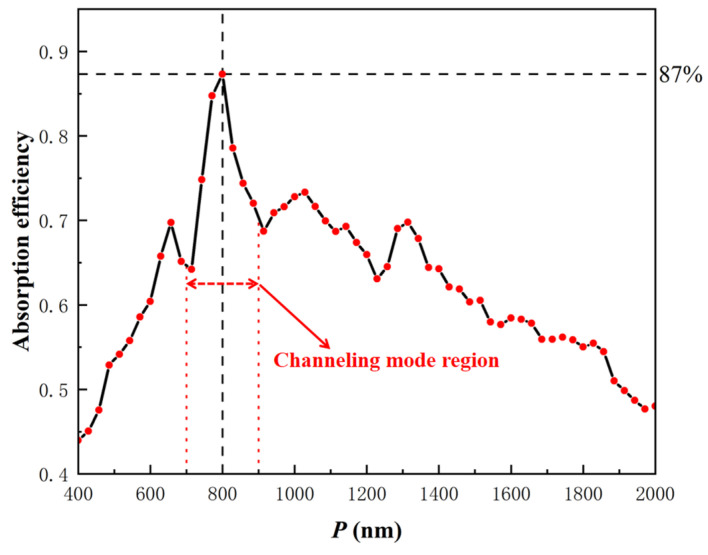
Simulated absorption efficiency at *λ* = 1.31 μm by scanning period, the red dot indicates the sampling point (2*R*/*P* = 0.7, *D* = 750 nm).

This work focuses on the design of a high responsivity InGaAs/Si APD for the 1310 nm wavelength. The design starts by optimizing the period *P* of the PT structure. Several past studies have indicated that the absorption efficiency can reach the maximum when the ratio of the hole diameter to the period (2*R*/*P*) is generally between 0.6~0.8 [10,12,15,16,21,22]. Therefore, we first set 2*R*/*P* to 0.7, and *D* was set at 750 nm to maintain a sufficiently large etching depth to keep a high light conductivity, the reason why *D* was not set to 1000 nm to cover the entire absorption layer is that the channeling mode guides the most light downward to Si rather than in the InGaAs absorption layer [23,24]. Based on the above parameters, we optimized the design to obtain the most suitable period *P* for the InGaAs/Si PT-APD; the result is shown in Figure 2.

When the period *P* is less than the incident wavelength *λ*, the channeling mode is generated in the PT structure, and the generation of the channeling mode is conducive to strengthening the optical coupling between the incident light and the model. The short and long wavelength edge of the channeling mode can be obtained by grating theory: the first reflected diffraction order (in SiO_2_) cuts-off at *λ* =$\;n_{\mathrm{SiO}2}P$; thus, for longer wavelengths only the specular reflection order is supported, leading to low reflection; the long wavelength edge coincides with the cut-off of the channeling mode, which is associated with the second diffraction order of the PT array [24]. The spectrum that produces the channeling mode can be estimated by the following inequality:

(1) $$n_{\mathrm{SiO}2}P<\lambda <Re\left(n_{eff}\right)P/\sqrt{2}$$

where $n_{eff}$ is an appropriate effective refractive index of the PT structure, according to Maxwell–Garnett theory, for cylindrical inclusions:

(2) $$n_{eff}=\sqrt{\varepsilon_{b}\frac{\varepsilon_{i}\left(1+f\right)+\varepsilon_{b}\left(1-f\right)}{\varepsilon_{i}\left(1-f\right)+\varepsilon_{b}\left(1+f\right)}}$$

where *f* is the inclusion (SiO_2_) volume fraction, and $\varepsilon_{i}$, $\varepsilon_{b}$ are the dielectric constants of the inclusion and background material, respectively [25]. According to the estimation of Equation (2), $Re\left(n_{eff}\right)$ is roughly equal to 2.64, then it can be concluded that the value of the period *P* is preferably in the region of 0.535 *λ* < *P* < 0.69 *λ.* Consequently, for the 1310 nm wavelength, the absorption efficiency can be kept in a high range when *P* = 700~900 nm in theory. As shown in Figure 2, *P* = 700~900 nm is the range with a high absorption efficiency; the simulation results in Figure 2 are highly matched with the theoretical value. Ultimately, the results show that the absorption layer can achieve a high absorption efficiency of 87% when the period *P* is equal to 800 nm, and 800 nm is chosen as the period *P* of the PT array.

Here, it is necessary to outline the theory about the photon-trapping structure. The photon-trapping array supports a set of modes with the wave vector in the Z direction (k_z_) and the wave vector (k_c_) in the X-Y plane. The array forms different collective modes based on different parameters, similar to the modes in photonic crystals. The research in photonic crystals has shown that slow light modes can appear under specific parameters, which is helpful to achieve very high absorption [26]. The APD diameter is generally larger than the thickness of the i-InGaAs layer; thus, increasing the k_c_ wave vector helps to lengthen the light propagation path and trap the light in the absorption layer. For this work *p* < *λ*; the model can support fundamental modes with k_c_ = 0 (mainly the Fabry–Perot resonant cavity mode), channeling modes that travel through the nano-holes, and guided-resonance modes that mostly exist in the high-index region between the SiO_2_ passivation layer and the SOI [9,24].

**Figure 3:**
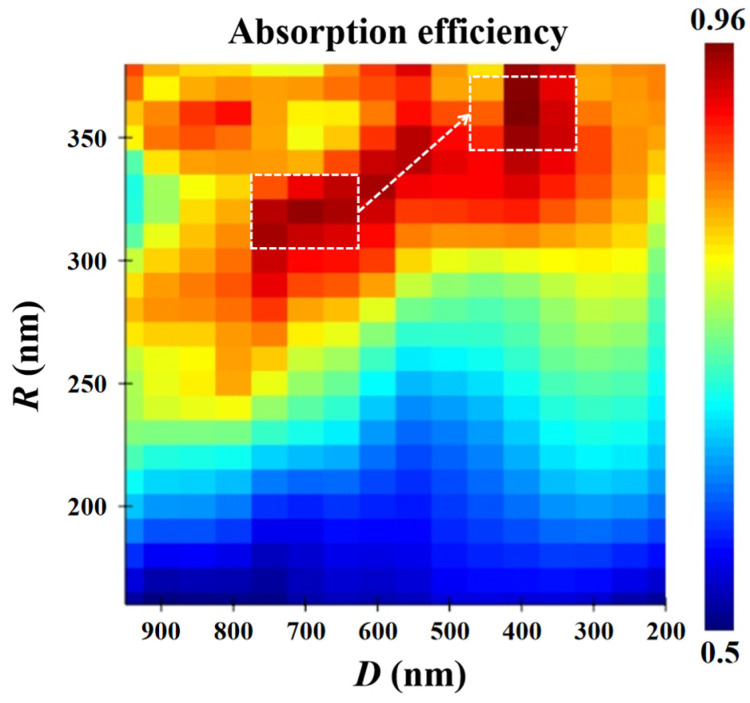
Two-dimensional maps of the absorption efficiency in terms of hole depth and hole radius at *λ* = 1.31 μm.

In order to further improve the absorption efficiency of PT-APD, the absorption efficiency of InGaAs/Si APD with the PT structure at *λ* = 1.31 μm was simulated by simultaneously scanning the hole radius (*R*) and hole depth (*D*) as shown in Figure 3, and the PT array period (*P*) was initially set to 800 nm. Considering that *R* and *D* cannot be set too small or too large, the scanning range of *R* was set between 160 nm and 380 nm, the scanning range of *D* was set between 200 nm and 950 nm. The corresponding simulation results are shown in Figure 3. The white dashed box indicates two regions with high absorption efficiency, and the highest absorption efficiency can reach more than 95%, which shows that the PT structure can obviously contribute to the improvement in the absorption of InGaAs/Si APD.

It can be seen from Figure 3 that the high-absorption-efficiency region gradually moves from the lower left to the upper right. Existing literature shows that the two-dimensional nano-hole array structure also can be used as a reflector with high reflectivity based on the guided mode resonance (GMR) effect [8], and because the period *P* is smaller than the incident wavelength in this work, this means that the main contribution to the absorption enhancement is the fundamental mode [24], so the above phenomenon can be mainly explained by the relevant principle of the Fabry–Perot (F-P) resonant cavity effect. In addition, the PT structure will produce the guided modes and GMR effect, the GMR effect is a coupling effect between grating diffraction and guided modes [27,28]; the guided modes are completely confined by the slab without any coupling to external radiations [29]. To sum up, the PT array cannot only act as a grating coupler [24], but also acts as a mirror to play a role in adjusting the resonant wavelength. 

For the original device, due to the large refractive index contrast between the semiconductor material and SiO_2_, the upper SiO_2_ and the SOI wafer act as top and bottom reflectors, respectively, and the constructed vertical cavity can effectively confine the incident light. Owing to multiple light reflections, the incident light has multiple absorption paths within the i-InGaAs layer, an amplified optical field is formed when the incident wavelength is just at the resonance wavelength, thereby improving the APD responsivity. At resonance wavelengths, the round-trip phase shift within the F-P cavity satisfies the following equation in the ideal perfect mirror condition:

(3) $$\frac{4\pi nL}{\lambda }=2\pi m$$

where *L* is the thickness of the cavity, *n* is the overall effective refractive index of the filling material, and *m* = 1,2,…. However, it is different when the PT array structure exists in the device. Under the same period *p*, a larger hole radius will produce a relatively small FF and $n_{eff}$, and the decreasing $n_{eff}$ causes a wider mode profile which results in a delayed phase at the SiO_2_/InGaAs bottom interface [8].

According to the effective medium theory, the effective refractive index of the PT structure depends on its fill factor (FF), which is defined as follows [30]:

(4) $$\mathrm{FF}=1-\left(\frac{\pi R^{2}}{P^{2}}\right)\;$$

where *R* and *P* are the hole radius and period of the PT structure, respectively. Therefore, for the optical model with a PT structure, Equation (3) no longer applies to this structure, and it is more accurate to use the following simple formula:

(5) $$\frac{4\pi nL}{\lambda }=2\pi m+\left|\varphi_{Delay}\right|$$

Assuming that *D* = 700 nm, *R* = 320 nm as the reference point, $\varphi_{Delay}$ is the delayed phase shift relative to the reference point. When *R* > 320 nm, the hole radius is larger than the reference point, $\left|\varphi_{Delay}\right|$ and it rises with the increase of the radius *R*. Since the wavelength of 1310 nm is fixed and only the length of the cavity can be adjusted accordingly, the increase in the cavity length is reflected by reducing the hole etching depth *D* to maintain the enhanced state of the resonator cavity.

**Figure 4:**
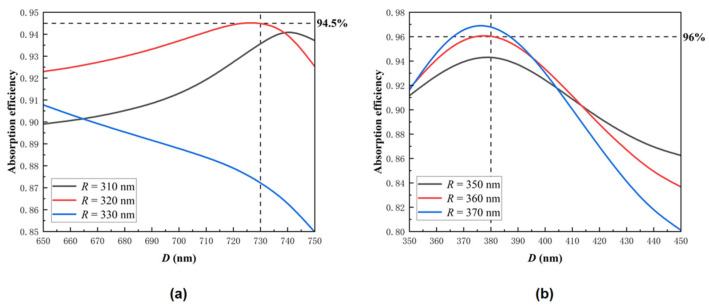
(**a**) The absorption efficiency of APD as a function of hole depth *D* = 650~750 nm when *R* = 310, 320, 330 nm; (**b**) the absorption efficiency of APD as a function of hole depth *D* = 350~450 nm when *R* = 350, 360, 370 nm.

The sampling interval of the etching depth *D* in Figure 3 is still relatively large. Therefore, we further optimize the hole etching depth *D* of the PT structure, and the results are shown in Figure 4. The results demonstrate that the PT structure allows a certain manufacturing tolerance, and the absorption efficiency can be maintained at least above 80% if the PT structure parameters cannot reach the theoretical values in the actual manufacturing process, which exhibits excellent fabrication-error robustness.

**Figure 5:**
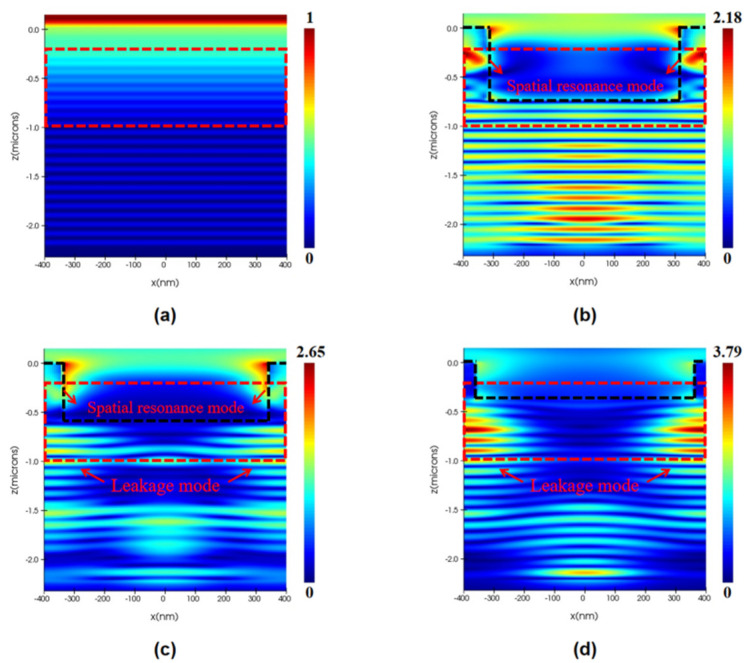
The Poynting vector distribution of the APD with (**a**) original structure, (**b**) *D* = 730 nm *R* = 320 nm PT structure, (**c**) *D* = 570 nm *R* = 340 nm PT structure, and (**d**) *D* = 380 nm *R* = 360 nm PT structure in the Z direction (the absorption layer is marked by the red dotted line, the SiO_2_ hole is above the black dotted line).

In order to further discuss the optical mechanism of the device, we studied the Poynting light energy distribution in the Y = 0 plane of the model, as shown in Figure 5. The red dashed box is the area where the absorbed energy is recorded, which is also where the i-InGaAs absorption layer is located. The PT structure can be regarded as a grating coupler and the underlying InGaAs and Si layers can be regarded as a waveguide layer, because the InGaAs material has strong absorption at the 1310 nm wavelength, most of the incident light will be absorbed in the i-InGaAs layer. The dynamic behavior of the GMR effect in the model is usually as follows: in the initial stage of power penetrating into the PT structure, the incident wave tends to concentrate in the grating and further diffracts into the waveguide layer; in the second stage, the optical power constrained in the waveguide layer forms a leakage mode and couples into neighboring gratings; in the stable stage, the leakage mode is periodically extended in the PT structure and partly converted into the spatial resonance mode [22,31].

Figure 5a exhibits the optical field distribution of a typical F-P cavity because the resonator cavity effect can be naturally generated in the SOI wafer-based InGaAs/Si APD. In Figure 5b, the main contribution to the light absorption are the spatial resonance mode and the fundamental mode under the PT structure, which means that most of the leakage mode in InGaAs layer is transformed into the spatial resonance mode; in Figure 5c, there are three obvious modes of spatial resonance mode, and the leakage mode and fundamental mode exist simultaneously in the device; in Figure 5d, the absorption enhancement is contributed mainly by the leakage mode and fundamental mode. When the hole radius *R* approaches the period *P*, the long wavelength light cannot be confined in the absorption layer between the nano-holes, resulting in a continued high-leakage-mode intensity in the InGaAs layer.

**Figure 6:**
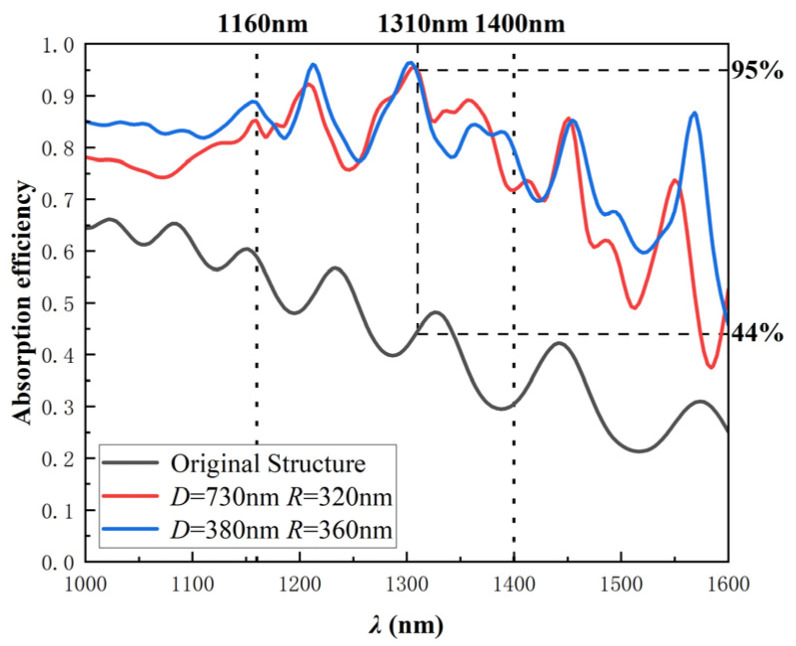
The absorption efficiency of InGaAs/Si APD as a function of the incident wavelength *λ* = 1~1.6 μm under different PT-structure conditions.

According to the grating correlation theory, in this design, a guided wave can be excited if the effective waveguide refractive index *N* =$\;\left|m\lambda /P\right|$ (for vertical incidence) is in the range [31]:

(6) $$n_{\mathrm{SiO}2}\leq \left|m\lambda /P\right|<n_{g}$$

where *m* is the diffraction order, $n_{\mathrm{SiO}2}\;$= 1.45, $n_{g}$ is the average index of the waveguide layer and is approximate to 3.5. Based on inequality (6), our design allows first-order diffracted light of *λ* > 1400 nm, first- and second-order diffracted light of *λ* = 1160~1400 nm, and second-order diffracted light of *λ* < 1160 nm coupling into the guided modes. In conclusion, as shown in Figure 6, for *λ* < 1160 nm, the total reflection angle of the second-order diffracted light in the waveguide is large, which makes it difficult for the guided mode to leak out and there is no strong resonance peak; for *λ* > 1400 nm, because the PT structure can act as both a strong refractive-index-modulation grating and a reflector, this means that the first-order diffracted light can exhibit the strong features of the F-P cavity effect and it is easier to leak out from the waveguide to form the GMR effect; for *λ* = 1160~1400 nm, combining the characteristics of the first-order and second-order diffracted light in the guided mode, the formant has a stronger absorption efficiency.

It can be seen from Figure 6 that the global absorption efficiency of PT-APD is much larger than that of the original structure APD, which indicates that all wavelengths of light can be coupled into the guided mode to a certain extent, thereby enhancing the broadband absorption. For *λ* = 1160~1400 nm, because both first- and second-order diffracted light can be coupled into the guided mode to form the GMR effect, the complex absorption spectrum in this band can be predicted. Overall, the absorption spectrum of PT-APD presented in Figure 6 can be concluded as smooth Fabry–Perot features are superimposed to sharp-guided resonance modes [24]. In addition, the PT structure also lessens the device’s junction area to reduce the junction capacitance, which helps to reduce the resistance-capacitance (RC) delay and improve the bandwidth characteristics of the APD [32,33].

**Figure 7:**
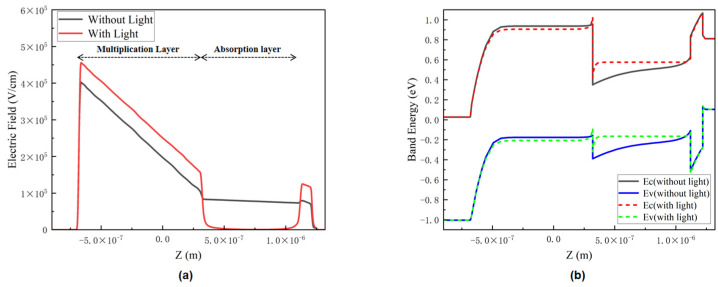
(**a**) Electric field distribution of the original InGaAs/Si APD at the same operating voltage along the vertical direction; (**b**) band structure of the original InGaAs/Si APD at 0 V.

In this work, we also conducted electrical simulations for the stacked structure in Figure 1b to investigate the electrical performances that InGaAs/Si APD should exhibit. By analyzing the electric field distribution of the APD at the operating voltage under illumination and without illumination, it is found that the internal electric field distribution is completely different with and without light, as shown in Figure 7a. Under the condition of without illumination, the multiplication layer of APD exhibits a maximum electric field intensity of 4 × 10^5^ V/cm, which meets the requirement of a high electric field in the multiplication layer, and the absorption layer can reach an electric field intensity of about 1 × 10^5^ V/cm to meet the requirements of maintaining the high-electron-saturated drift velocity; under the condition of with illumination, the electric field of the absorption region is exceedingly low, which will lead to the low drift velocity of the current carrier, this phenomenon may be detrimental to the application of InGaAs/Si APD in a high-speed optical communication field. 

The present semiconductor band structure theory showed that the accumulation of electric charge also has a great influence on the bending of the energy band. The energy band structure comparison at 0 V bias is shown in Figure 7b, the band structure in the absence of light condition is in line with our expectations, but in the presence of light, the curved potential well and potential barrier appeared concurrently at the heterojunction interface. The bend of the energy-band structure can be attributed to the fact that a large number of photo-generated carriers will be generated in the absorption layer under illumination condition, and the photo-generated electrons will accumulate at the interface of InGaAs/Si heterojunction to form the space-charge effect due to the large conduction band offset ΔEc = 0.6 eV between InGaAs and Si, which is reflected in the bending of the energy band. Eventually, the electric field opposite to the applied electric field caused by the space charge effect leads to the phenomenon of an extremely low electric field in the absorption region. In addition, the blocking of holes by p-InP layer in the valence band also plays a significant role in the above phenomenon.

**Figure 8:**
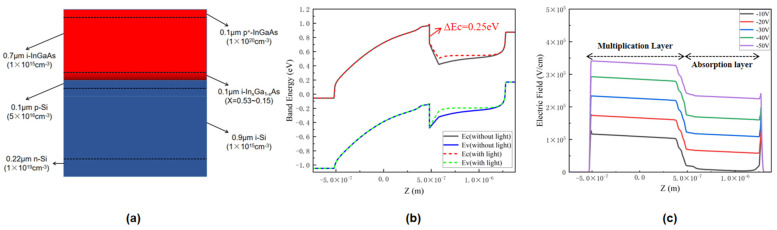
(**a**) The stacked structure diagram of SAGCM-InGaAs/Si APD. (**b**) Band structure of the SAGCM-InGaAs/Si APD at 0 V. (**c**) Electric field distribution of the SAGCM-InGaAs/Si APD at different bias voltages along the vertical direction under illumination condition.

In order to make InGaAs/Si APD overcome the above shortcomings, we proposed a separate absorption, grading, charge, and multiplication (SAGCM) InGaAs/Si APD based on the original stacked structure, as shown in Figure 8a. In this new stack structure, the Ga content in the i-InGaAs grading layer is graded from 0.47 to 0.85 to relieve the conduction band offset between InGaAs and Si [5], and the p-Si charge layer is used to regulate the electric field of multiplication and absorption layers. In Figure 8b, it can be found that there is no great bending in the energy-band structure with and without illumination, which indicates that the new structure can effectively overcome the accumulation of electrons near the heterojunction. The reason can be attributed to the addition of the In_x_Ga_1-x_As grading layer between i-InGaAs and p-Si, which reduces the large conduction-band offset ΔEc from 0.6 eV to 0.25 eV. From the electric field distribution at different voltages in Figure 8c, it can be seen that the electric field intensity of the multiplication layer and the absorption layer increase with the raising of the bias voltage, and the highest electric field intensity of the multiplication layer can reach about 3.4 × 10^5^ V/cm.

**Figure 9:**
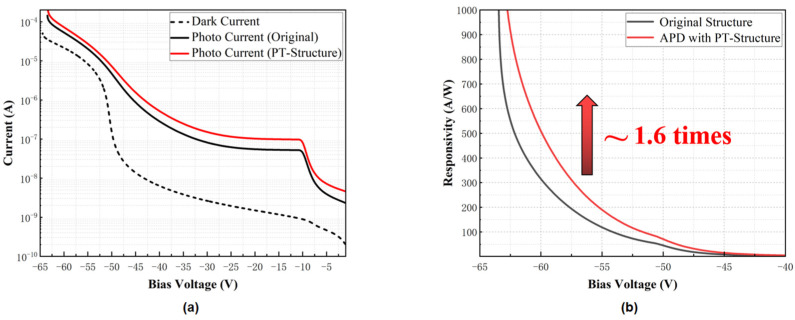
The simulated (**a**) I-V curve and (**b**) responsivity of the SAGCM-InGaAs/Si APD with and without PT structure (the mesa-size of APD is 8 μm × 8 μm, and the input optical power is 100 nW).

Finally, the simulated dark current, photo current, and the responsivity of the optimized InGaAs/Si APD as a function of the bias voltage is shown in Figure 9, the responsivity is defined as the ratio of photo-generated current (photo current minus dark current) to optical input power. The electrical model simulates the electrical characteristics of the 8-μm mesa-size APD, and the optical input power is set to 100 nW. In Figure 9b, it can be found that the responsivity of PT-APD is larger than that of APD without PT structure, and in the linear operation mode of InGaAs/Si-APD, the responsivity of PT-APD is about 1.6 times that of the original structure APD at the same operating voltage, which corresponds to the enhancement of the absorption efficiency of the optimized device.

## 4. Conclusions

In summary, we designed a PT structure combined with an InGaAs/Si APD based on a SOI wafer, which can realize a higher responsivity at the linear operation mode. In order to make it possible to apply the device in the field of communication, an optimized SAGCM-InGaAs/Si APD was proposed. Compared with the original device, the absorption performance of the PT-APD could be greatly improved by optimizing the PT-structure parameters, and the responsivity of the PT-APD was enhanced by 1.6 times at the same operating voltage. This work explored a new technical feasibility for high-sensitivity, low-noise optical communication devices.

## Data Availability

Not applicable.
